# Unconventional Electrochemistry in Micro-/Nanofluidic Systems

**DOI:** 10.3390/mi7050081

**Published:** 2016-05-03

**Authors:** Sahana Sarkar, Stanley C. S. Lai, Serge G. Lemay

**Affiliations:** MESA+ Institute for Nanotechnology, University of Twente, P.O. Box 217, 7500 AE Enschede, The Netherlands; s.sarkar-1@utwente.nl (S.S.); s.c.s.lai@utwente.nl (S.C.S.L.)

**Keywords:** electrochemistry, reference electrode, bipolar electrode, floating electrode, potentiometry

## Abstract

Electrochemistry is ideally suited to serve as a detection mechanism in miniaturized analysis systems. A significant hurdle can, however, be the implementation of reliable micrometer-scale reference electrodes. In this tutorial review, we introduce the principal challenges and discuss the approaches that have been employed to build suitable references. We then discuss several alternative strategies aimed at eliminating the reference electrode altogether, in particular two-electrode electrochemical cells, bipolar electrodes and chronopotentiometry.

## 1. Introduction

One of the main challenges in creating micro- and nanodevices for chemical analysis is downscaling the measurement system that is ultimately used for readout. Several features of electrochemistry render it a desirable mechanism for transducing chemical information into electrical signals [1,2,3,4,5,6,7,8,9,10,11,12,13,14,15]: The fabrication of electrodes suitable for electrochemistry is largely compatible with the methods employed for creating micro- and nanofluidic channels, it requires minimal additional (relatively low-cost) equipment, its sensitivity often increases with the downscaling of the electrode dimensions, it directly yields electrical signals without an intermediary transduction step (e.g., light), and it operates at relatively low power. Nonetheless, electrochemical methods can prove challenging to implement in micro- and nanosystems: While the concepts and instrumentation required for such measurements are well developed on the macroscopic scale, subtle, unobvious adjustments and compromises are often necessary upon downscaling. This complexity often goes unrecognized in the design of miniaturized systems, limiting accuracy and performance. 

The aim of this review is to introduce the key concepts that influence electrochemical measurements in micro- and nanoscale measurement systems. Our target audience consists of scientists and engineers working on miniaturizing electrochemical measurement systems. We assume that the reader is already familiar with the methods used to fabricate micro-/nanofluidic devices and with basic electrochemical principles [16,17], and concentrate on elucidating some of the key factors that influence electrochemical measurements in miniature systems. We pay particular attention to how the electrostatic potentials of electrodes are established, determined, and controlled - or not, as is often the case. We first discuss reference electrodes, a key component of most macroscopic electrochemical measurement systems. This allows introducing the notation used in the reminder of the article as well as some important concepts that are sometimes misunderstood. We then discuss two classes of systems in which the conventional electrode biasing scheme is abandoned, namely, electrochemical cells without a reference electrode and bipolar electrodes. We end with a brief discussion of potentiometric measurements, in which the potential of an electrode is not controlled but is instead employed for detection. Unless stated otherwise, we assume that the test solution consists of water containing both redox-active analyte molecules as well as a much higher concentration of inert salt ions, the so-called supporting electrolyte. This situation is typical for, e.g., biomedical samples. We concentrate on fluidic devices and exclude individual miniature electrodes used in conjunction with macroscopic measurement cells, conventional electrodes modified with nanomaterials, and electrochemical scanning probe techniques, which are reviewed extensively elsewhere [18,19,20,21].

## 2. Anatomy of an Electrode 

**Figure 1:**
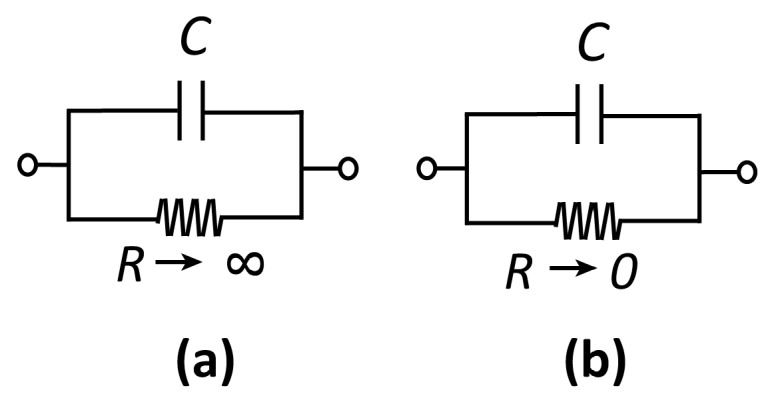
Equivalent circuits for (**a**) a polarizable and (**b**) a non-polarizable interface.

Before discussing specific electrochemical systems, we introduce a few key concepts that will recur throughout this review[1]. The interface between a solution (an ionic conductor) and an electrode (an electronic conductor, typically a metal, but also potentially a semiconductor or a macromolecule) can be represented by a capacitor *C* and a (nonlinear) resistor *R* in a parallel configuration, as shown in Figure 1. Here, *C* represents the buildup of charge in the so-called electrical double layer (EDL) that develops at this interface. The EDL consists of electrons (or holes) in the electrode and compensating ions in the solution. These lead to an electric field—and thus an electrostatic potential difference—between the solution and the electrode. The EDL is highly local, for example, extending only on the order of ~1 nm for water at physiological concentrations. The resistor *R*, on the other hand, represents the transfer of electrons between the electrode and the redox species in solution via electrochemical reactions.

Electrodes can be qualitatively classified as polarizable or non-polarizable. In the case of a polarizable electrode, *R* is very high and it is therefore possible to alter the potential difference across the interface without injecting significant current into the measurement cell. On the contrary, if *R* is very low, changing the potential difference across the capacitor requires the application of very large currents, as charge is “leaked” through the interface. This short-circuit-like behavior is referred to as a non-polarizable interface. In practice, no electrode is ever fully polarizable or non-polarizable; whether an electrode represents a good approximation to either depends on the magnitude of the voltages and currents that occur in a particular measurement.

## 3. Reference Electrodes 

**Figure 2:**
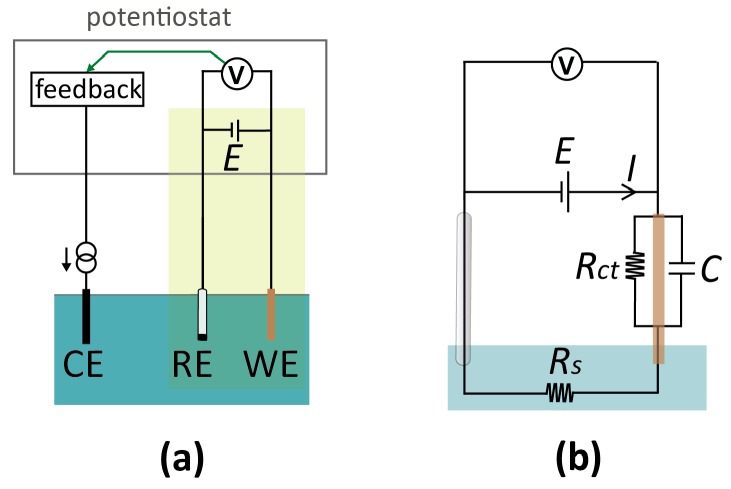
(**a**) Schematic of a conventional electrochemical cell for voltammetric measurement. The cell consists of three electrodes, termed the working (WE), reference (RE), and counter electrode (CE), immersed in the electrolyte solution. A potential, *E*, is applied to the WE with respect to the RE. If the current through the RE would be high enough to cause a potential shift, a CE is introduced to minimize the current through the RE. At low currents, it is instead possible to operate with a two-electrode configuration and eliminate the CE altogether (highlighted in green), simplifying the detection circuitry. (**b**) Equivalent circuit diagram of a two-electrode setup. *R_s_*: solution resistance; *R_ct_*: charge-transfer resistance at the WE; *C:* electrical double layer capacitance at the WE. This circuit treats the RE as ideally non-polarizable.

In macroscopic systems, electrochemical measurements are typically carried out in a three-electrode configuration [16], as shown schematically in Figure 2a. The working (or indicator) electrode (WE) is the electrode where the analytical measurement takes place: An electrochemical reaction occurs if the potential difference between this electrode and the adjacent solution is such as to favor electron transfer, leading to a current. This electrode is coupled to an electrode of a known, defined potential, called the reference electrode (RE). The (conceptual) circuit diagram of this two-electrode system is depicted in Figure 2b. Importantly, potentials applied to the WE are always with respect to the potential of the RE. Thus, an RE provides a reference point for the potential (similar to the role of ground in electronic circuits). However, it is important to note that the actual electrostatic potential difference between the RE and the solution may not be (and, in practice, rarely is) zero, and one therefore needs to specify the type of RE when stating the voltage of a WE (e.g., “1 V *vs.* Ag/AgCl (3 M KCl)” for a silver/silver chloride reference electrode immersed in a 3 M potassium chloride solution). Similarly, an often overlooked nuance is that applying an *external* potential of 0 V with respect to the RE does not insure that no potential difference exists between the WE and the adjacent solution. 

Any electrode system can serve as an RE as long as it approaches ideal non-polarizability, meaning that its interfacial potential remains essentially fixed with the passage of currents [16,22]. The amount of current that can pass depends on the specific RE system and design, but in general non-polarizability breaks down at “high” currents [22], and the reference potential will vary (for a commercial, macroscopic RE, this is typically in the order of µA’s). Consequently, the WE potential is not controlled accurately at high currents, as a (undefined and variable) part of the applied potential between the WE and RE, *E*, is dropped at the RE-electrolyte interface. To circumvent this issue, one can introduce a third electrode, the counter (or auxiliary) electrode (CE). In this three-electrode setup, the current from the WE is routed through the CE, which acts as the electron source or sink for the reaction at the WE. The terminal controlling the RE has a high input impedance, rendering the current drawn through the RE negligible, and the RE interfacial potential thus remains constant. The technical implementation for potential control and current measurement in a three-electrode setup employs a potentiostat. Conceptually, this instrument monitors the potential difference between WE and RE, which is used as a feedback signal to control the current passing through the CE so that the actual potential difference matches the desired (applied) potential difference. A detailed description of the workings of a potentiostat can be found in many textbooks on electrochemistry and electrochemical instrumentation [16,23]. As a final note, it should be borne in mind that a CE (and potentiostat by extension) is only required if the current in the system is large, and may be bypassed in miniaturized sensors if currents of the order of a few µA are measured that can be directly passed through a RE without significantly affecting its potential. In our experience, this condition is easily satisfied in most micro- and nanoscale systems. This results in compact simplified electronics, shown by the yellow box in Figure 2a, which essentially consists of a power source and an ammeter connected in series with the two electrodes.

**Solution resistance.** While in principle the RE only sets the electrostatic potential near its surface, the solutions employed in electrochemical measurements are ionic conductors. As a result, the potential of a solution when no electrical current is flowing through it is uniform throughout its entire bulk volume and is set by the RE. An important exception occurs at the boundaries of the liquid, where EDLs can develop as discussed above. This is particularly relevant near the surface of the WE, where a potential difference is required to drive electrochemical processes. However, if a net current, *I*, is flowing through the solution, an electric field can develop according to Ohm’s law (*E* = *IR_s_*, where *R_s_* is the solution ionic resistance), and part of the applied voltage is dropped in the solution between the RE and WE. These ohmic voltage drops can be minimalized either by reducing the current (e.g., by decreasing the analyte concentration or reducing the size of the electrode) or by minimizing the electrolyte resistance between the RE and WE (e.g., by increasing the conductivity of the electrolyte solution or placing the RE close to the WE to decrease the length of the resistive path). In most electroanalytical measurements, the analyte concentration is much lower than the electrolytic (salt) concentration; therefore, these ohmic voltage drops may reasonably be neglected. However, if an electrolytic solution of low conductivity (usually due to low ionic strength) is used, *IR_s_* may be significant and needs to be taken into account when considering the WE potential (*E_WE_* = *E* − *IR_s_*). This can be particularly significant in fluidic devices where confinement of the liquid easily leads to higher values of *R_s_* than is typical in macroscopic experiments. 

**Requirements.** At this point, it is worth discussing the technical requirements of a reference electrode. A RE should have a potential which is stable over time [22] and which is not significantly altered by small perturbations to the system—in particular, the passage of a small current. Some of the main considerations while designing a RE are discussed in depth by Shinwari *et al.* [22]. Commercial REs typically employ a macroscopic piece of metal (providing an “infinite” reservoir of redox species) coated with a sparingly soluble metal salt (such that the interfacial concentration is determined by the solubility product of the salt), immersed in a contained reference solution, and the entire system is connected to the test solution by a salt bridge (to prevent composition changes of the reference solution while minimizing the liquid junction potential) [16,24]. While such electrode systems are straightforward to realize on the macroscale, implementing REs in miniaturized systems requires careful considerations in the downscaling of all these components [22,25]. 

**Miniaturized REs.** Several analogues to conventional REs have been demonstrated using microfabrication, and several techniques are available for their manufacture such as thin film deposition [26,27,28,29,30], electroplating [31,32], or screen printing [33,34] of the metal followed by ion exchange reactions or electrochemical coating. The interface to the test solution and reference solution chamber is typically implemented using gels or nanoporous membranes/glass. For example, an Ag/AgCl electrode was replicated by a thin-film deposition of Ag supported over Pt, after which AgCl was formed by oxidizing it in a solution containing chloride ions [31]. In another example, miniaturization of the liquid junction Ag/AgCl was demonstrated by covering a deposited thin film of silver with a layer of polyamide. This layer had a slit at the center where AgCl was grown; the liquid junction was formed with photo-curable hydrophilic polymer [35]. 

However, the stability of such miniaturized references electrodes is often limited, and typical problems include limited lifetimes, poor reproducibility, and drifting electrode potentials [22,36]. A common cause is the rapid consumption of the electrode material due to its small size. In general, electrode consumption can be divided into an electrochemical (Equation (1)) and a chemical (Equation (2)) pathway.

AgCl (s) + e^−^ ⇌ Ag (s) + Cl^−^ (aq) (electrochemical) (1)

AgCl (s) + *n*Cl^−^ (aq) ⇌ AgCl_(*n*+1)_*^n^*^−^ (aq), where 0 < *n* < 3 (chemical) (2)

In the electrochemical pathway, the passage of a small current through a miniaturized RE can already be sufficient to induce complete consumption of the electrode material within experimental time scales. For example, a microscopic Ag/AgCl RE of an area of 100 µm^2^ (AgCl thickness 100 nm) exposed to a current of only 10 pA would be completely consumed within approximately one hour. The chemical pathway relates to the non-zero solubility of the metal salt, where the dissolved and solid species are only in chemical equilibrium as long as the solution is saturated with the metal salt. If the RE is exposed to a non-saturated solution, or the solution is continuously replenished (such as in flow systems), dissolution of the metal salt will occur. This issue is further exacerbated in the case of Ag/AgCl electrodes, where there is a non-negligible formation of aqueous AgCl_(*n*+1)_*^n^*^−^ ion complexes in chloride-containing solutions [37,38]. At physiological electrolyte concentrations, this leads to an equilibrium concentration of dissolved AgCl in the µM range, sufficient to completely dissolve a 100 µm^2^ × 100 nm AgCl layer in ~0.1 µL of electrolyte solution. 

Another common cause for the limited stability of miniaturized REs is the possible contamination of the reference solution via non-ideal (“leaky”) bridging membranes. This issue can be alleviated by eliminating the salt bridge and reference solutions. Such systems are commonly termed quasi- or pseudo-RE. While the terms are often used interchangeably, there is a subtle but important difference between the two. A quasi-RE simply omits the reference solution and immerses the electrode directly into the test solution [28,29,39,40,41,42,43,44,45]. A clearly defined redox couple, however, sets the electrode potential, and any fluctuations result from changes in the activity coefficients of this couple. For example, a common Ag/AgCl quasi-RE consists of a silver electrode coated with silver chloride salt and in contact with the chloride-containing test solution; here, the Ag/Ag^+^ couple sets the solution potential [30]. On the other hand, a pseudo-RE refers to a large surface area electrode (such as a platinum or silver wire) directly exposed to the solution [42,43,45]. In this case, which redox couple sets the reference potential is undefined, and the reference potential remains reasonably constant by virtue of the large surface area, with even low reactivity being sufficient to take up small currents without significant polarization of the electrode. In both cases, the RE can be calibrated by measuring its potentials relative to a conventional RE. Thus, while miniaturizing REs still present challenges, rational design can provide a microscopic RE which is sufficiently stable given the requirements for a specific measurement.

Finally, it is worthwhile to consider the placement of electrodes in microfabricated systems. In a macroscopic system, the CE is placed far from the WE and RE, such that the substances produced at the CE do not reach the WE surface to interfere with the measurements there. However, in microscopic systems, this might not be possible due to space requirements, and such interference needs to be taken into account in order to avoid undesirable shifts in the reference potential. 

## 4. Systems without a Reference Electrode

Considering the difficulties inherent in implementing miniaturized high-quality reference electrodes, it is natural that considerable effort has been devoted to creating analytical systems in which the role of the reference is minimized or omitted altogether. Doing so comes at a price since in such cases the interfacial potentials that drive electron-transfer reactions at the system’s electrodes is no longer explicitly controlled. As a result, no universally applicable alternative to the conventional combination of potentiostat and reference electrode has evolved. Nonetheless, reliable alternatives can be implemented in some particular geometries and/or when sufficient information about the solution to be analyzed is available. 

**Figure 3:**
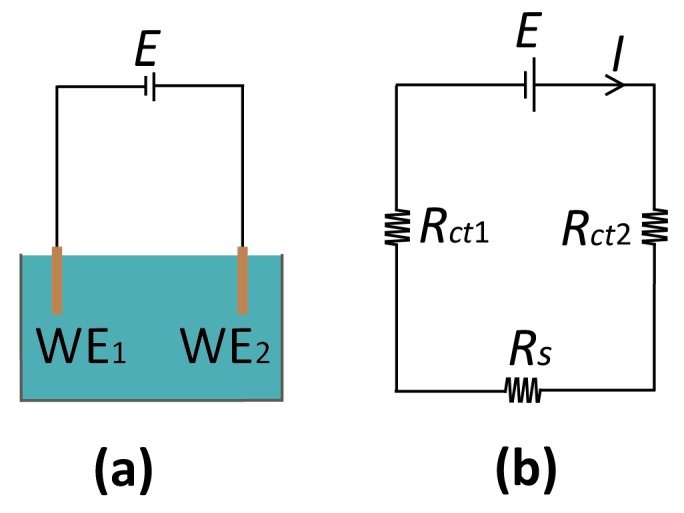
(**a**) Reference-less two-electrode system where *E* is the applied potential between the two WEs. (**b**) Corresponding equivalent-circuit diagram. *R_s_*: solution resistance; *R_ct_*_1,2_: (charge transfer) resistance at the WE_1,2_.

The basic configuration for a reference-free, two-electrode system is sketched in Figure 3. While this represents the simplest case of a system without an RE, the discussion of the solution potential in the following is general, and can be extended to incorporate additional electrode elements. The most important feature of the system of Figure 3 is that the interfacial potential differences at the two electrodes is not controlled separately since only the total potential difference between the two electrodes is accessible experimentally. The potential of the bulk electrolyte phase, *E_s_*, is thus instead free to float to different values. This is in stark contrast with the case where one of the electrodes is an RE; in that case, there is no change in the potential difference at the RE interface, and the potential of the electrolyte is pinned to the RE potential.

What sets the potential of the solution in the experiment of Figure 3? The passage of a current at one of the electrodes causes charge to be injected in this solution. As discussed above in the context of reference electrodes, this charge accumulates at the boundaries of the bulk phase. For example, an oxidation reaction taking place at an electrode causes the withdrawal of electrons from the solution and the accumulation of positive charge at its boundaries, in turn causing the electrostatic potential of the solution to become more positive. This acts as a negative feedback mechanism, as the shift in solution potential acts to inhibit the electrochemical process that caused it (in our example, the oxidation current decreases by making the solution more positive with respect to the electrode). The solution eventually settles to a stationary steady state at a potential such that no net charge injection takes place, that is, the total current being injected into the solution vanishes:

(3) $$\sum_{j}I_{j}=0,\text{ where }I_{j}=\;I_{j}\left(V_{j}-E_{s}\right)$$

here, *I_j_* is the current through the *j*th electrode, which is a function of its interfacial potential difference (*V_j_ – E_s_*), *V_j_* is the potential applied to the electrode, and *E_s_* is the solution potential (neglecting ohmic drops for ease of notation) with respect to a common reference point in the circuit such as signal ground. In principle, if the relations between current and interfacial potential at each of the electrodes are known (because, e.g., they can be derived from fundamental electrochemical kinetic theory or they have been experimentally determined), then it is possible to solve for the unique value of *E_s_* that satisfies Equation (3) and to deduce the current through each of the electrodes. This procedure essentially amounts to solving the equivalent circuit shown in Figure 3b, where the electrochemical reactions are represented by (highly nonlinear) resistors *R_ct_*_1_ and *R_ct_*_2_, and Equation (3) is the direct application of Kirchhoff’s current law.

For the two-electrode system of Figure 3a, Equation (3) reduces to the statement that the solution potential will shift in such a way that the reduction current at the more negative of the two electrodes is equal in magnitude to the oxidation current at the more positive electrode. This scenario was discussed in detail by Xiong and White [46], where it was, for example, shown explicitly that increasing the area of one of the electrodes causes the solution potential to shift closer to that electrode’s open-circuit potential because that electrode’s effective resistance becomes smaller.

A further consequence of Equation (3) is that parasitic pathways for a current—such as may result from a minor leak—can sometimes have a significant influence in a microsystem without a reference electrode. In conventional electrochemical cells, such a parasitic current can be accommodated by the counter electrode (or the reference for low-current systems) without influencing the signal measured at the working electrode. For a floating solution potential, however, even relatively small uncompensated currents can lead to drift. This was illustrated by Sarkar *et al.* [47], who showed how the (large) redox-cycling current between two electrodes separated by 65 nm can be controlled by the (much smaller) current to an additional electrode located outside the nanofluidic device [47].

## 5. Bipolar Electrodes 

**Figure 4:**
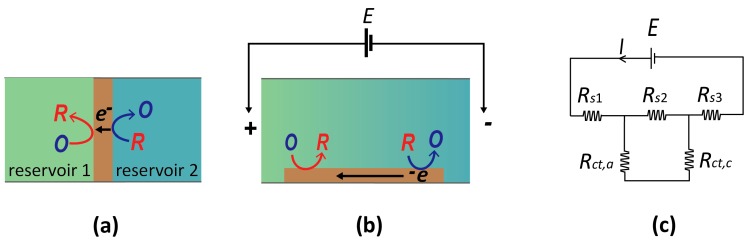
(**a**) Schematic diagram of a bipolar electrode (brown) in contact with two separate reservoirs. (**b**) Alternative concept of a bipolar electrode in which a uniform electric field is applied along a channel filled with electrolytic solution. A band electrode exposed to this solution exhibits bipolarity at its opposing ends (cathodic at left and anodic on right). (**c**) Equivalent circuit for panel (b). *E* is the potential applied across the solution, *R_s_* is the resistance of the solution, and *R_ct_* is the charge transfer resistance across the anodic/cathodic ends of the bipolar electrode (BPE).

A bipolar electrode (BPE) is a *floating* conductor which facilitates opposing electrochemical reactions (oxidizing and reducing) on spatially separated regions of its surface. Two example systems are shown in Figure 4. Figure 4a represents the (conceptually) simplest case. Here, two electrolyte solutions are physically separated by a BPE, such that the only current path between them is through the BPE. Since it is a good conductor, the entire BPE is essentially at the same potential, while the relative potential of the two electrolyte solutions can be changed independently. Consequently, the local interfacial potential difference of the BPE with the adjacent solution is different at the two ends. If suitable species are present in the two reservoirs, reduction and oxidation processes may occur at the two ends of a BPE, thereby coupling two, otherwise isolated, electrochemical systems.

Alternatively, a BPE can be located in a single reservoir (Figure 4b). Two additional electrodes are then placed at the ends of the reservoir, and applying a large current between them induces an electric field in the electrolyte due to its finite conductivity (ohmic drop). As shown in Figure 4b, this spatially heterogeneous solution potential leads to a gradient of electrostatic potential differences along the length of the BPE (that is, between the electrode and the solution). If a sufficiently large potential difference between the two ends of the bipolar electrode is induced, it becomes possible to drive an oxidation reaction at one end and a reduction at the opposite end of the same electrode, similarly to the case of Figure 4a. 

From a purely conceptual point of view, the scenarios shown in Figure 3a and Figure 4b are very closely related. In each case, one element of the electrochemical circuit—the solution in Figure 3a and the BPEs in Figure 4—is free to adjust its electrostatic potential in response to redox reactions taking place at spatially separated regions. It is therefore unsurprising that the same basic principles apply for determining the potential to which the BPE drifts in response to electrochemistry. In fact, Equation (1) carries over directly to this case, where now *E_s_* represents the potential of the bipolar electrode, and *j* is an index that runs over the different regions of this electrode (for the case of a continuous gradient as in Figure 4b, the sum becomes an integral over the electrode surface, but the underlying principle remains unchanged). 

The defining feature of BPEs is that they are floating electrodes, yet can be induced to facilitate electrochemical reactions of choice at their interface. This is particularly attractive for miniaturized systems, as abolishing the need for contacts to solution (*i.e.*, reference electrodes) simplifies fabrication and instrumentation. Furthermore, it enables an arbitrarily large number of BPEs (such as arrays of BPEs imbedded in insulating matrices [48]) to be driven simultaneously. The use of BPEs in the micro-/nanoscopic domain was pioneered by Bradley *et al.* [49], who demonstrated the use of bipolar electrochemistry to create electrical contacts in microcircuits by employing copper electrodeposition as the cathodic reaction. This work was followed by a dramatic increase in the investigation of bipolar electrochemistry—in particular, by the groups of Kuhn [50,51,52,53,54,55] and Crooks [56,57,58,59]. A recent review by Sequeira *et al.* [60] discusses bipolar electrochemistry and their many varied applications that several contemporary groups are presently exploring. As a particularly striking example, Mallouk, Sen, and colleagues [61,62,63] demonstrated a locomotion mechanism for bipolar microswimmers based on electrochemical reactions taking place at both ends of the swimmer. Another intriguing variant is to use the bipolar electrode to couple the reaction of a target analyte to a second, separate reaction that produces an optically active species. Using the latter’s fluorescent properties allowed for the demonstration of the highly sensitive, fluorescence-mediated detection of species that are not themselves optically active [48,64]. 

**Implicit bipolar behavior.** Apart from devices that explicitly exploit bipolar electrochemistry as their mode of operation, this effect has an important consequence for the design and validation of electrochemical detection devices. Any conductor in contact with solution has the potential to act as a bipolar electrode if its potential is not controlled. This is a very different situation from conventional electronic devices, where leaving a particular component unconnected typically means that it can be safely ignored, at best, or a source of stray capacitance, at worse. A well-documented example of a system where bipolar electrochemistry is implicitly utilized is scanning electrochemical microscopy (SECM in the positive feedback mode), where a conducting sample can be left unbiased but then acts as a bipolar electrode [65,66]. Similarly, floating electrodes imbedded in nanochannels were shown to act as “short circuits” to a reference located outside the nanofluidic device [47,67]. Last but not least, it is important to keep in mind that all solvents—especially water—are liable to electrochemical breakdown; if a sufficient potential gradient is applied, any floating metal features in a device can become implicated in reactions involving water, protons, hydroxide, or dissolved oxygen, leading to unintended currents flowing through the system [68,69].

## 6. Potentiometry 

The main theme of this review has been the control of potentials in electrochemical systems. For completeness, we discuss here very briefly potentiometry, the branch of analytical chemistry concerned with the measurement of potentials as a detection mechanism. It is difficult to understate the importance of potentiometry as it forms the basis for many widely used technologies, starting with pH-sensitive electrodes and extending to a wide family of other ion-selective electrodes [70,71,72,73]. 

In its most common form, potentiometry is an equilibrium technique, with the potential of a working electrode being measured with respect to a reference. This makes it particularly sensitive to the choice of RE, which becomes challenging to implement in miniaturized systems given all the complications discussed above. Commonly used for concentration determination, lower detection limits of such techniques can be achieved with downscaling, and extensive work has been carried out in the development of so-called nanopotentiometry. Much of this work has focused on nanostructured thin films interfaced to macroscopic electrodes [70,72]. To what extent these approaches and materials can be adapted in the context of, e.g., lithographically fabricated micro- and nanodevices largely remains an interesting question for future work. Thus, while this is an area where we expect major developments will likely happen in the near future, we do not attempt to discuss specific works at this time. 

**Figure 5:**
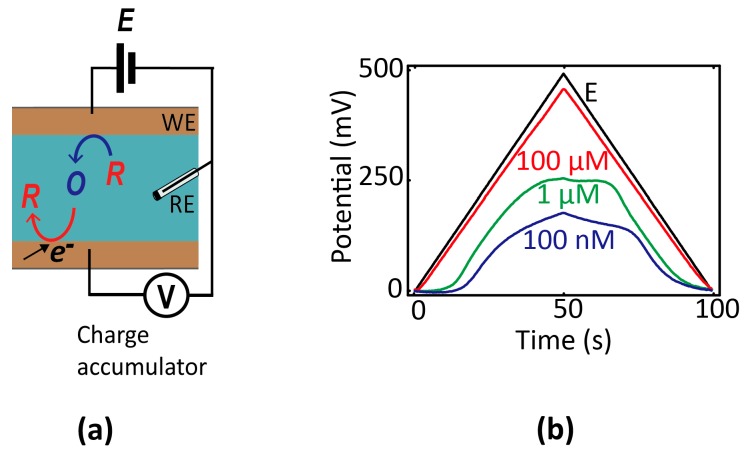
(**a**) Schematic diagram of a two-electrode nanogap system in contact with a solution containing reversible redox species. The bottom (unbiased) electrode accumulates charge over time, and the resulting potential shift is used as readout signal. (**b**) Chronopotentiometric signal *versus* concentration of redox species (100 µM, 10 µM, and 1 nM Fc(MeOH)_2_ in 0.1 M KCl) in response to a triangular potential wave applied to the top electrode (black line).

One variant that may lend itself more readily to integrated miniature systems is so-called chronopotentiometry [12], in which the potential of an electrode is monitored as a function of time using high-impedance readout circuitry. Before equilibrium is established, electrochemical reactions occurring at an electrode cause its potential to shift over time. The rate of change of the potential is proportional to the electrochemical current and inversely proportional to the electrode capacitance; hence, a concentration can in principle be extracted from the time-dependent data. To explicitly illustrate this principle within the nanogaps [47], we show in Figure 5 measurements of the potential of a floating electrode over time as it accumulates charge due to redox cycling in a nanofluidic device (consisting of two electrodes, one of which is floating). The evolution of the potential over time reflects the rate of electrochemical charge transfer, which itself depends on the composition of the solution.

Furthermore, since small electrodes normally have lower capacitances, the potentiometric signal is more sensitive in this case, making the method a logical candidate for miniaturization. Based on the additional consideration that the readout of potentials is relatively straightforward to implement in conventional complementary metal-oxide semiconductor (CMOS) electronics [74], Zhu *et al.* [75,76,77] suggested that chronopotentiometry is particularly well suited for systems in which fluidics and electronics are implemented on a single, highly integrated chip. Whether this type of electrochemical analysis can offer a competitive alternative to existing methods is presently an open question.

## 7. Summary and Outlook

The emergence of point-of-care diagnostic systems has led to the rapprochement of micro-/nanofluidics and electrochemical sensing methods, and this trend can be expected to strengthen in the coming years. Although electrochemistry is an exhaustive subject and a vast amount of information is available in the literature, it is not always straightforward for new researchers in the area of microsystems to identify the concepts and approaches that are most relevant for building practical miniaturized devices. This is particularly true because some standard ingredients of electrochemical analysis—especially the use of optimized reference electrodes—are surprisingly challenging to scale down. While universally applicable solutions have yet to emerge, many common pitfalls can be avoided by informed experimental design. We have thus attempted to provide an introduction to the methods of micro-/nanoelectrochemistry and, in particular, to make the reader aware of non-idealities which are not necessarily obvious when extrapolating from the macro domain. We hope that linking some of the concepts addressed in this paper will be beneficial to the fluidic sensor community and will help to stimulate further exploration of the rich field of miniature sensing technologies.

## References

[1] Oja S.M., Fan Y.S., Armstrong C.M., Defnet P., Zhang B. (2016). Nanoscale electrochemistry revisited. Anal. Chem..

[2] Oja S.M., Wood M., Zhang B. (2013). Nanoscale electrochemistry. Anal. Chem..

[3] Watkins J.J., Zhang B., White H.S. (2005). Electrochemistry at nanometer-scaled electrodes. J. Chem. Educ..

[4] Rackus D.G., Shamsi M.H., Wheeler A.R. (2015). Electrochemistry, biosensors and microfluidics: A convergence of fields. Chem. Soc. Rev..

[5] Rassaei L., Singh P.S., Lemay S.G. (2011). Lithography-based nanoelectrochemistry. Anal. Chem..

[6] Meier J., Schiotz J., Liu P., Norskov J.K., Stimming U. (2004). Nano-scale effects in electrochemistry. Chem. Phys. Lett..

[7] Arrigan D.W.M. (2004). Nanoelectrodes, nanoelectrode arrays and their applications. Analyst.

[8] Kang S., Lemay S.G., Mirkin M.V., Amemiya S. (2015). Nanoelectrochemical methods. Nanoelectrochemistry.

[9] Micheal M.V., Amemiya A. (2015). Nanoelectrochemistry.

[10] Lee T.M.H. (2008). Over-the-counter biosensors: Past, present, and future. Sensors.

[11] Bakker E., Qin Y. (2006). Electrochemical sensors. Anal. Chem..

[12] Grieshaber D., MacKenzie R., Voros J., Reimhult E. (2008). Electrochemical biosensors—Sensor principles and architectures. Sensors.

[13] Ino K. (2015). Microchemistry- and MEMS-based integrated electrochemical devices for bioassay applications. Electrochemistry.

[14] Wei D., Bailey M.J.A., Andrew P., Ryhanen T. (2009). Electrochemical biosensors at the nanoscale. Lab Chip.

[15] Gencoglu A., Minerick A.R. (2014). Electrochemical detection techniques in micro- and nanofluidic devices. Microfluid. Nanofluid..

[16] Bard A.J., Faulkner L.R. (2000). Electrochemical Methods: Fundamentals and Applications.

[17] Brett C.M.A., Brett A.M.O. (1993). Electrochemistry: Principles, Methods, and Applications.

[18] Moore A.M., Weiss P.S. (2008). Functional and spectroscopic measurements with scanning tunneling microscopy. Annu. Rev. Anal. Chem..

[19] Mirkin M.V., Nogala W., Velmurugan J., Wang Y.X. (2011). Scanning electrochemical microscopy in the 21st century. Update 1: Five years after. Phys. Chem. Chem. Phys..

[20] Sun P., Laforge F.O., Mirkin M.V. (2007). Scanning electrochemical microscopy in the 21st century. Phys. Chem. Chem. Phys..

[21] Macpherson J.V., Unwin P.R. (2001). Noncontact electrochemical imaging with combined scanning electrochemical atomic force microscopy. Anal. Chem..

[22] Shinwari M.W., Zhitomirsky D., Deen I.A., Selvaganapathy P.R., Deen M.J., Landheer D. (2010). Microfabricated reference electrodes and their biosensing applications. Sensors.

[23] Hickling A. (1942). Studies in electrode polarisation part IV—The automatic control of the potential of a working electrode. Trans. Faraday Soc..

[24] Brezinski D.P. (1983). Kinetic, static and stirring errors of liquid junction reference electrodes. Analyst.

[25] Mousavi M.P.S., Buhlmann P. (2013). Reference electrodes with salt bridges contained in nanoporous glass: An underappreciated source of error. Anal. Chem..

[26] Kim T.Y., Hong S.A., Yang S. (2015). A solid-state thin-film Ag/AgCl reference electrode coated with graphene oxide and its use in a pH sensor. Sensors.

[27] Webster T.A., Goluch E.D. (2012). Electrochemical detection of pyocyanin in nanochannels with integrated palladium hydride reference electrodes. Lab Chip.

[28] Matsumoto T., Ohashi A., Ito N. (2002). Development of a micro-planar Ag/AgCl quasi-reference electrode with long-term stability for an amperometric glucose sensor. Anal. Chim. Acta.

[29] Uludag Y., Olcer Z., Sagiroglu M.S. (2014). Design and characterisation of a thin-film electrode array with shared reference/counter electrodes for electrochemical detection. Biosens. Bioelectron..

[30] Rivas I., Puente D., Ayerdi I., Castano E. Ag/AgI quasi-reference microelectrodes. Proceedings of the 2005 Spanish Conference on Electron Devices.

[31] Zhou J.H., Ren K.N., Zheng Y.Z., Su J., Zhao Y.H., Ryan D., Wu H.K. (2010). Fabrication of a microfluidic Ag/AgCl reference electrode and its application for portable and disposable electrochemical microchips. Electrophoresis.

[32] Polk B.J., Stelzenmuller A., Mijares G., MacCrehan W., Gaitan M. (2006). Ag/AgCl microelectrodes with improved stability for microfluidics. Sens. Actuator B Chem..

[33] Da Silva E.T.S.G., Miserere S., Kubota L.T., Merkoci A. (2014). Simple on-plastic/paper inkjet-printed solid-state Ag/AgCl pseudoreference electrode. Anal. Chem..

[34] Desmond D., Lane B., Alderman J., Glennon J.D., Diamond D., Arrigan D.W.M. (1997). Evaluation of miniaturised solid state reference electrodes on a silicon based component. Sens. Actuator B Chem..

[35] Suzuki H., Shiroishi H., Sasaki S., Karube I. (1999). Microfabricated liquid junction Ag/AgCl reference electrode and its application to a one-chip potentiometric sensor. Anal. Chem..

[36] Suzuki H. (2000). Advances in the microfabrication of electrochemical sensors and systems. Electroanalysis.

[37] Du J.L., Chen Z.F., Chen C.C., Meyer T.J. (2015). A half-reaction alternative to water oxidation: Chloride oxidation to chlorine catalyzed by silver ion. J. Am. Chem. Soc..

[38] Fritz J.J. (1985). Thermodynamic properties of chloro-complexes of silver-chloride in aqueous-solution. J. Solut. Chem..

[39] Da Silva R.A.B., de Almeida E.G.N., Rabelo A.C., da Silva A.T.C., Ferreira L.F., Richter E.M. (2009). Three electrode electrochemical microfluidic cell: Construction and characterization. J. Braz. Chem. Soc..

[40] Simonis A., Dawgul M., Luth H., Schoning M.J. (2005). Miniaturised reference electrodes for field-effect sensors compatible to silicon chip technology. Electrochim. Acta.

[41] Franklin R.K., Johnson M.D., Scott K.A., Shim J.H., Nam H., Kipke D.R., Brown R.B. Iridium oxide reference electrodes for neurochemical sensing with MEMS microelectrode arrays. Proceedings of IEEE Sensors 2005.

[42] Beati A.A.G.F., Reis R.M., Rocha R.S., Lanza M.R.V. (2012). Development and evaluation of a pseudoreference Pt//Ag/AgCl electrode for electrochemical systems. Ind. Eng. Chem. Res..

[43] Kasem K.K., Jones S. (2008). Platinum as a reference electrode in electrochemical measurements. Platin. Met. Rev..

[44] Dacuna B., Zaragoza G., Blanco M.C., Quintela A.L., Mira J., Rivas J. (1998). Electrochemical synthesis of Fe/Ag and Co/Ag granular thin films. Mater. Sci. Forum.

[45] Yang H.S., Kang S.K., Choi C.A., Kim H., Shin D.H., Kim Y.S., Kim Y.T. (2004). An iridium oxide reference electrode for use in microfabricated biosensors and biochips. Lab Chip.

[46] Xiong J.W., White H.S. (2013). The I-V response of an electrochemical cell comprising two polarizable microelectrodes and the influence of impurities on the cell response. J. Electroanal. Chem..

[47] Sarkar S., Mathwig K., Kang S., Nieuwenhuis A.F., Lemay S.G. (2014). Redox cycling without reference electrodes. Analyst.

[48] Oja S.M., Zhang B. (2014). Imaging transient formation of diffusion layers with fluorescence-enabled electrochemical microscopy. Anal. Chem..

[49] Bradley J.C., Chen H.M., Crawford J., Eckert J., Ernazarova K., Kurzeja T., Lin M.D., McGee M., Nadler W., Stephens S.G. (1997). Creating electrical contacts between metal particles using directed electrochemical growth. Nature.

[50] Loget G., Li G.Z., Fabre B. (2015). Logic gates operated by bipolar photoelectrochemical water splitting. Chem. Commun..

[51] Loget G., Zigah D., Bouffier L., Sojic N., Kuhn A. (2013). Bipolar electrochemistry: From materials science to motion and beyond. Acc. Chem. Res..

[52] Loget G., Roche J., Gianessi E., Bouffier L., Kuhn A. (2012). Indirect bipolar electrodeposition. J. Am. Chem. Soc..

[53] Loget G., Kuhn A. (2012). Bipolar electrochemistry for cargo-lifting in fluid channels. Lab Chip.

[54] Fattah Z., Loget G., Lapeyre V., Garrigue P., Warakulwit C., Limtrakul J., Bouffier L., Kuhn A. (2011). Straightforward single-step generation of microswimmers by bipolar electrochemistry. Electrochim. Acta.

[55] Loget G., Kuhn A. (2010). Propulsion of microobjects by dynamic bipolar self-regeneration. J. Am. Chem. Soc..

[56] Scida K., Sheridan E., Crooks R.M. (2013). Electrochemically-gated delivery of analyte bands in microfluidic devices using bipolar electrodes. Lab Chip.

[57] Chang B.Y., Chow K.F., Crooks J.A., Mavre F., Crooks R.M. (2012). Two-channel microelectrochemical bipolar electrode sensor array. Analyst.

[58] Sheridan E., Hlushkou D., Anand R.K., Laws D.R., Tallarek U., Crooks R.M. (2011). Label-free electrochemical monitoring of concentration enrichment during bipolar electrode focusing. Anal. Chem..

[59] Dumitrescu I., Anand R.K., Fosdick S.E., Crooks R.M. (2011). Pressure-driven bipolar electrochemistry. J. Am. Chem. Soc..

[60] Sequeira C.A.C., Cardoso D.S.P., Gameiro M.L.F. (2016). Bipolar electrochemistry, a focal point of future research. Chem. Eng. Commun..

[61] Wang Y., Hernandez R.M., Bartlett D.J., Bingham J.M., Kline T.R., Sen A., Mallouk T.E. (2006). Bipolar electrochemical mechanism for the propulsion of catalytic nanomotors in hydrogen peroxide solutions. Langmuir.

[62] Kline T.R., Paxton W.F., Mallouk T.E., Sen A. (2005). Catalytic nanomotors: Remote-controlled autonomous movement of striped metallic nanorods. Angew. Chem. Int. Ed..

[63] Paxton W.F., Kistler K.C., Olmeda C.C., Sen A., St Angelo S.K., Cao Y.Y., Mallouk T.E., Lammert P.E., Crespi V.H. (2004). Catalytic nanomotors: Autonomous movement of striped nanorods. J. Am. Chem. Soc..

[64] Ma C.X., Zaino L.P., Bohn P.W. (2015). Self-induced redox cycling coupled luminescence on nanopore recessed disk-multiscale bipolar electrodes. Chem. Sci..

[65] Oleinick A.I., Battistel D., Daniele S., Svir I., Amatore C. (2011). Simple and clear evidence for positive feedback limitation by bipolar behavior during scanning electrochemical microscopy of unbiased conductors. Anal. Chem..

[66] Richter M.M. (2004). Electrochemiluminescence (ECL). Chem. Rev..

[67] Zevenbergen M.A.G., Wolfrum B.L., Goluch E.D., Singh P.S., Lemay S.G. (2009). Fast electron-transfer kinetics probed in nanofluidic channels. J. Am. Chem. Soc..

[68] Arora A., Eijkel J.C.T., Morf W.E., Manz A. (2001). A wireless electrochemiluminescence detector applied to direct and indirect detection for electrophoresis on a microfabricated glass device. Anal. Chem..

[69] Leinweber F.C., Eijkel J.C.T., Bower J.G., van den Berg A. (2006). Continuous flow microfluidic demixing of electrolytes by induced charge electrokinetics in structured electrode arrays. Anal. Chem..

[70] Bakker E., Pretsch E. (2007). Modern potentiometry. Angew. Chem. Int. Ed..

[71] Malon A., Vigassy T., Bakker E., Pretsch E. (2006). Potentiometry at trace levels in confined samples: Ion-selective electrodes with subfemtomole detection limits. J. Am. Chem. Soc..

[72] Bakker E., Pretsch E. (2008). Nanoscale potentiometry. Trends Anal. Chem.

[73] Pungor E., Toth K. (1970). Ion-selective membrane electrodes—A review. Analyst.

[74] Singh P.S. (2015). From sensors to systems: CMOS-integrated electrocheimcal biosensors. IEEE Access.

[75] Zhu X.S., Choi J.W., Ahn C.H. (2004). A new dynamic electrochemical transduction mechanism for interdigitated array microelectrodes. Lab Chip.

[76] Zhu X.S., Ahn C.H. (2005). Electrochemical determination of reversible redox species at interdigitated array micro/nanoelectrodes using charge injection method. IEEE Trans. Nanobiosci..

[77] Zhu X.S., Ahn C.H. (2006). On-chip electrochemical analysis system using nanoelectrodes and bioelectronic CMOS chip. IEEE Sens. J..

